# hPaf1/PD2 interacts with OCT3/4 to promote self-renewal of ovarian cancer stem cells

**DOI:** 10.18632/oncotarget.14775

**Published:** 2017-01-20

**Authors:** Saswati Karmakar, Parthasarathy Seshacharyulu, Imayavaramban Lakshmanan, Arokia P. Vaz, Seema Chugh, Yuri M. Sheinin, Sidharth Mahapatra, Surinder K. Batra, Moorthy P. Ponnusamy

**Affiliations:** ^1^ Department of Biochemistry and Molecular Biology, University of Nebraska Medical Center, Omaha, NE, USA; ^2^ Fred and Pamela Buffett Cancer Center, Eppley Institute for Research in Cancer and Allied Disease, University of Nebraska Medical Center, Omaha, NE, USA; ^3^ Department of Pathology and Microbiology, University of Nebraska Medical Center, Omaha, NE, USA; ^4^ Department of Pediatrics, University of Nebraska Medical Center, Omaha, NE, USA

**Keywords:** hPaf1/PD2, CSC, ovarian cancer, OCT3/4, self-renewal

## Abstract

Cancer stem cells (CSCs), which mediate drug resistance and disease recurrence in several cancers, are therapeutically relevant to ovarian cancer (OC), wherein approximately 80% of patients manifest with tumor recurrence. While there are several markers for ovarian CSCs (OCSCs), the mechanism for their self-renewal maintenance by unique driver/markers is poorly understood. Here, we evaluated the role of hPaf1/PD2, a core component of RNA Polymerase II-Associated Factor (PAF) complex, in self-renewal of OCSCs through marker and functional analyses, including CRISPR/Cas9-silencing of hPaf1/PD2 in OCSCs and provided a possible mechanism for maintenance of OCSCs. Expression of hPaf1/PD2 showed moderate to intense staining in 32.4% of human OC tissues, whereas 67.6% demonstrated basal expression by immunohistochemistry analysis, implying that the minor proportion of cells overexpressing hPaf1/PD2 could be putative OCSCs. Isolated OCSCs showed higher expression of hPaf1/PD2 along with established CSC and self-renewal markers. Knockdown of hPaf1/PD2 in OCSCs resulted in a significant downregulation of CSC and self-renewal markers, and impairment of *in vitro* tumor sphere (*P* < 0.05) and colony formation (*P* = 0.013). Co-immunoprecipitation revealed that OCT3/4 specifically interacts with hPaf1/PD2, and not with other PAF components (Ctr9, Leo1, Parafibromin) in OCSCs, suggesting a complex-independent role for hPaf1/PD2 in OCSC maintenance. Moreover, there was a significant overexpression and co-localization of hPaf1/PD2 with OCT3/4 in OC tissues compared to normal ovary tissues. Our results indicate that hPaf1/PD2 is overexpressed in OCSCs and maintains the self-renewal of OCSCs through its interaction with OCT3/4; thus, hPaf1/PD2 may be a potential therapeutic target to overcome tumor relapse in OC.

## INTRODUCTION

Ovarian cancer (OC) has the highest mortality rate among gynecologic cancers in the USA [1]. Due to its asymptomatic nature, OC is often advanced or metastatic at the time of diagnosis, with up to 70% of patients presenting with extra-ovarian disease [1]. In these patients, treatment usually involves cisplatin and surgical debulking if disease permits. For patients who do respond to treatment, the majority of the patients still relapse within 6–16 months [2].

This relapse may be attributable to a small population of cancer cells that have the ability to self-renew and differentiate, commonly known as cancer stem cells (CSCs), which have been demonstrated in patients with OC along with other solid tumors [3–5]. CSCs exhibit dysregulated cellular pathways, and contribute to not only disease recurrence, but also drug resistance [3, 6]. This emphasizes the need to characterize this subset of cancer cells. It is known that OCSCs express markers such as CD133, CD44, CD24, CD117, ESA, and ALDH1 but the mechanisms responsible for OCSC self-renewal remain largely unknown [7, 8].

hPaf1 (human RNA Polymerase II-Associated Factor 1)/PD2 (Pancreatic Differentiation 2) is the human homolog of the yeast Paf1 and is a core component of the PAF (RNA Polymerase II-Associated Factor) complex [9]. The hPAF complex plays a critical role in the recruitment of RNA polymerase II to transcripts, thereby participating in mRNA elongation and 3’ end processing [10, 11]. Moreover, hPaf1/PD2 mediates post-translational histone modifications such as H3K4 di- and tri-methylation and H2B monoubiquitination, thereby affecting chromatin structure [12, 13]. In addition, PAF1 plays a gatekeeper role for RNA Polymerase II promoter-proximal pausing in metazoans, and its loss enhances the transcription of thousands of genes [14, 15]. Apart from PAF complex-dependent roles, several studies have documented that hPaf1/PD2 can function independent of the complex in specialized biological aspects of cancer, such as cell cycle progression, acinar to ductal metaplasia in pancreatic cancer, tumorigenicity, and metastasis [9, 16–18]. Another recent study has proposed that hPaf1/PD2 is a novel marker for pancreatic CSCs that mediates their drug resistance [19]. hPaf1/PD2 has also been shown to regulate the self-renewal process of mouse embryonic stem cells through its interaction with OCT3/4 [20]. However, the importance and specific function of hPaf1/PD2 expression in OC and its CSC counterpart has not been previously investigated.

In the present study, we investigated the involvement of hPaf1/PD2 in the maintenance of OCSCs. We observed that knockdown of hPaf1/PD2 resulted in downregulation of CSC and self–renewal markers with a concomitant loss of CSC phenotype. Further, CRISPR/Cas9-mediated silencing of hPaf1/PD2 in OC cells resulted in a significant reduction in percentage of OCSCs. In addition, we demonstrated the interaction of hPaf1/PD2 with OCT3/4 in OCSCs. Our results indicate that hPaf1/PD2 plays a major role in the maintenance of self-renewal of OCSCs through its interaction with OCT3/4.

## RESULTS

### hPaf1/PD2 is differentially expressed in human ovarian cancer tissues

**Figure 1:**
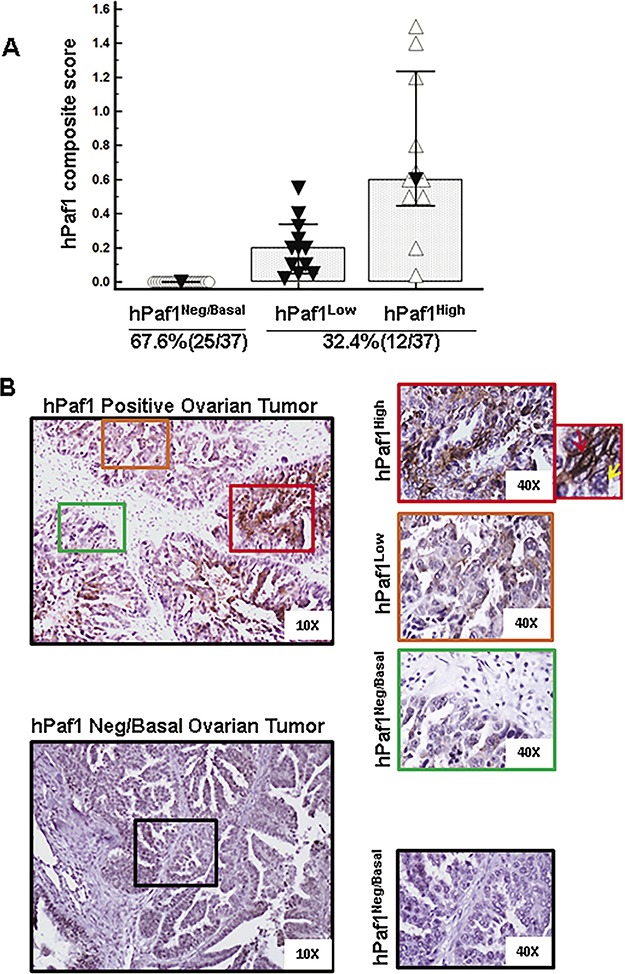
Expression of hPaf1/PD2 in human ovarian cancer tissues Expression of hPaf1/PD2 was evaluated in 37 human ovarian cancer tissues using immunohistochemistry. Tissues were categorized as hPaf1/PD2^negative/basal^ (intensity = 0), hPaf1/PD2^low^ (intensity = 1) and hPaf1/PD2^high^ (intensity > 1) on the basis of intensity of staining. The extent of hPaf1/PD2-positive staining in human ovarian cancer tissues was scored as actual percentages. A composite score (CS) was calculated by multiplying intensity and positivity, which ranges between 0 and 3. The majority of samples (67.6%) exhibited negative /basal expression of hPaf1/PD2, whereas 32.4% showed low-to-moderate and high expression. **(A)** Graphical representation of hPaf1/PD2 composite score versus categories of hPaf1/PD2 staining (hPaf1/PD2^negative/basal^, hPaf1/PD2^low^ and hPaf1/PD2^high^). **(B)** Representative images of an hPaf1/PD2 positive and an hPaf1/PD2^negative/basal^ ovarian tumor. Areas enclosed within boxes are magnified and represented on the right. Among the positive tissues, expression of hPaf1/PD2 ranged from hPaf1/PD2^high^ (box with red outline) to hPaf1/PD2^low^ (box with orange outline) to hPaf1/PD2^negative/basal^ (box with green outline). The topmost box on the extreme right represents a zoomed image wherein a cell with negative/basal hPaf1/PD2 expression is indicated by a yellow arrow and a cell with high expression is indicated with a red arrow.

Accumulating evidence suggests that chemoresistance and recurrence of OC is mediated by CSCs [3, 21]. In this study, we examined the expression pattern of hPaf1/PD2 in OC tissues with IHC and investigated its functional role in OCSCs. Based on the intensity of staining, tissues were categorized as hPaf1/PD2^negative/basal^ (intensity = 0), hPaf1/PD2^low^ (intensity = 1) and hPaf1/PD2^high^ (intensity > 1) (Figure 1A and Supplementary Table 1). We found that 67.6% (25 out of 37) of OC tissues showed negative/basal expression of hPaf1/PD2. Further, the remaining 32.4% (12 out of 37) tissues showed a differential pattern of hPaf1/PD2 staining, ranging from mild to moderate and intense expression (Figure 1B). Thus, only a sub-population of human OC tissues exhibited hPaf1/PD2 positive expression, implying that hPaf1/PD2 overexpressing cells could be putative OCSCs. This representation of hPaf1/PD2 in a minor population of OC tissues is in accordance with the ‘cancer stem cell model’ of tumor evolution [22]. However, basal expression of hPaf1/PD2 is required to perform normal biological functions such as transcription elongation and epigenetic modifications [10, 12].

### Isolation and characterization of OCSCs from OC cell lines

In order to determine if hPaf1/PD2 is indeed overexpressed in OCSCs, we isolated OCSCs or side population (SP) cells from two OC cell lines. Flow sorting using Hoechst 33342 staining revealed that OVCAR3 exhibited 3% SP cells, whereas A2780 showed 0.6% SP cells (Supplementary Figure 1). Isolated SP cells were cultured under CSC-specific conditions and enriched with cisplatin (IC_20_= 2 μM) treatment. Morphologically, SP/CSCs were very different from non-side population (NSP) cells: SP cells formed tight circular colonies referred to as ‘cobblestone structure,’ whereas NSP cells resembled differentiated cells (Supplementary Figure 2A).

With multiple rounds of cell divisions, the isolated SP cells can give rise to a heterogeneous population of CSCs and more differentiated progeny of CSCs by virtue of their properties of self-renewal and asymmetric division [23]. Given that CSCs are drug resistant, treatment with a chemotherapeutic agent such as cisplatin, which is lethal to differentiated cells, enriches CSCs [24]. This was validated with an observed morphological difference between SP cells, in the presence and absence of cisplatin. OVCAR3 SP cells treated with cisplatin formed a more ‘cobblestone–like’ structure compared to SP cells without cisplatin treatment (Supplementary Figure 2B). *In vitro* tumor sphere formation is a measure of self-renewal and tumorigenic potential of CSCs, which exploits the ability of CSCs to grow in a non-adherent culture and form tumor spheres. We observed a greater number and larger tumor spheres with SP cells isolated from OVCAR3 compared to NSP cells, which formed fewer and significantly smaller tumor spheres (*P* < 0.02) (Supplementary Figure 2C). These results indicate that the isolated SP cells represent a truly distinct population of OCSCs.

### hPaf1/PD2 is co-overexpressed with established CSC markers and self-renewal markers in SP compared to NSP cells

**Figure 2:**
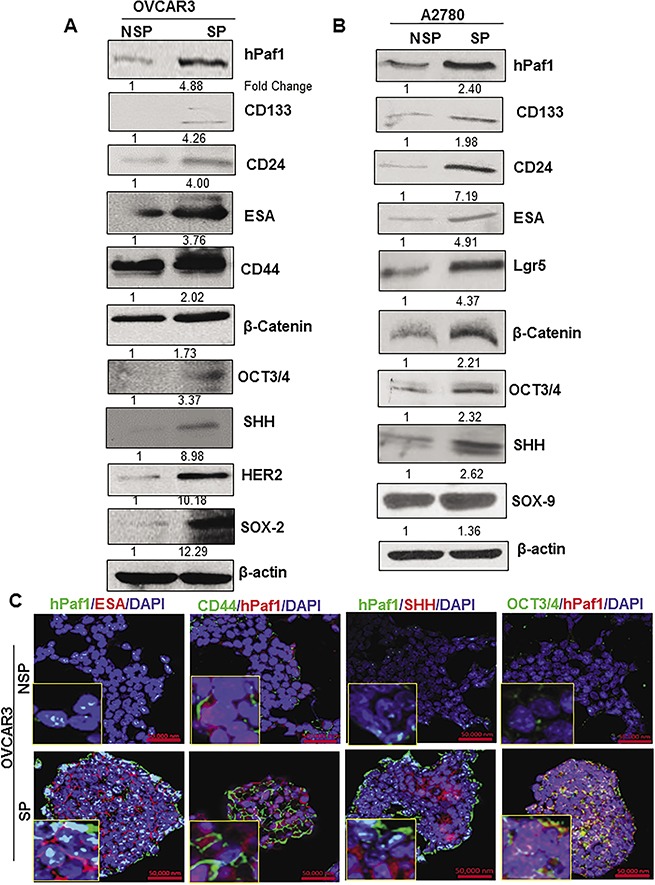
Expression of cancer stem cell markers and self-renewal markers in SP cells isolated from ovarian cancer cell lines There was a higher expression of CSC markers (CD133, CD24, ESA, CD44, Lgr5), and self-renewal markers (SHH, β-Catenin, OCT3/4, HER2, SOX-9 and SOX-2) in SP cells compared to NSP cells isolated from ovarian cancer cell lines **(A)** OVCAR3 **(B)** A2780 analyzed by Western blotting. An equal amount of protein was loaded in each well. β-actin was used as a loading control. **(C)** Confocal microscopic analysis revealed a greater expression of ESA, and CD44 (CSC markers) as well as SHH, and OCT3/4(self-renewal markers) along with hPaf1/PD2 in SP cells compared to NSP cells.

We observed that hPaf1/PD2 was significantly overexpressed in SP cells (OCSCs) isolated from OVCAR3 compared to NSP cells (non-OCSCs). There was also a higher expression of CSC markers such as CD133, CD44, CD24, and ESA, as well as self-renewal markers such as β-Catenin, SOX-2, OCT3/4, Sonic Hedgehog (SHH), and Epidermal growth factor family protein 2 (HER2) (Figure 2A). Similarly, hPaf1/PD2 was overexpressed in SP cells isolated from A2780 compared to NSP cells along with CSC markers such as CD133, CD24, ESA, Lgr5, and self-renewal proteins such as β-Catenin, SHH, OCT3/4, and SOX-9 by immunoblotting (Figure 2B). Through immunofluorescence analysis, we also found a significantly higher co-expression of hPaf1/PD2 with CSC markers (ESA, and CD44) and self-renewal proteins (OCT3/4, and SHH) in OVCAR3 SP cells compared to NSP cells (Figure 2C). Moreover, we observed co-localization of OCT3/4 with hPaf1/PD2 in OVCAR3 SP cells (Figure 2C). These results suggest that hPaf1/PD2 overexpressing SP cells are the putative OCSCs because they exhibit higher expression of known OCSC and self-renewal markers.

### Knockdown of hPaf1/PD2 affects the CSC phenotype

**Figure 3:**
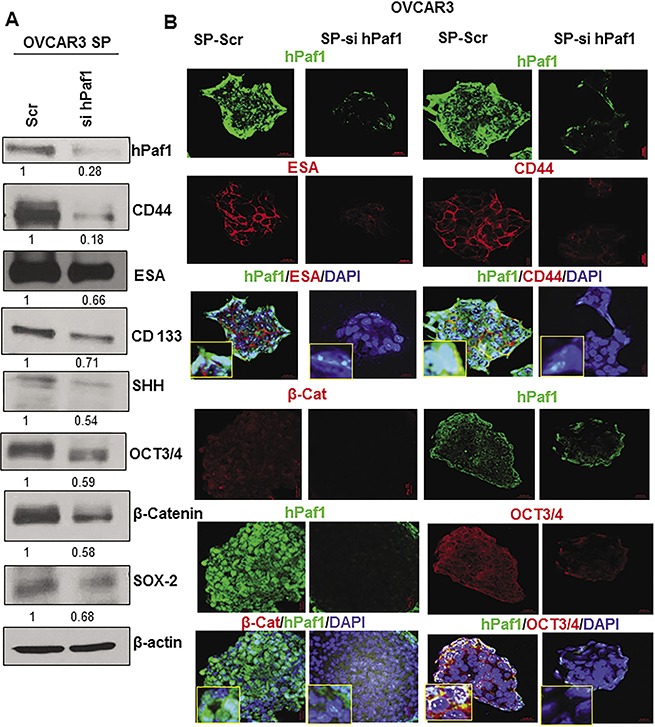
Effect of knockdown of hPaf1/PD2 on expression of established CSC and self-renewal markers Transient knockdown of hPaf1/PD2 was performed by transfecting 100 pmol of hPaf1/PD2 specific siRNA into OVCAR3 SP cells. Non-targeting siRNA (100 pmol) was used as a control. (**A**) Knockdown of hPaf1/PD2 resulted in decreased expression of CSC markers (CD44, ESA, and CD133) and self-renewal markers (SHH, β-Catenin, OCT3/4, and SOX-2) analyzed by Western blotting. Equal amount of protein was loaded in each well. β-actin was used as a loading control. (**B**) Confocal microscopic analysis revealed a significant decrease in expression of CD44 and ESA (CSC markers) on knockdown of hPaf1/PD2 (upper panels). Lower panels depict a marked decrease in expression of OCT3/4 and β-Catenin (self-renewal markers) with transient knockdown of hPaf1/PD2.

To investigate whether hPaf1/PD2 plays a role in the maintenance of OCSCs, we transiently knocked down hPaf1/PD2 in OVCAR3 SP cells using specific siRNA. We observed around 80% knockdown of hPaf1/PD2 in SP cells (Figure 3A), and this knockdown resulted in a significant reduction in expression of CSC markers (CD44, CD133, and ESA) as well as of self-renewal proteins (SHH, β-Catenin, OCT3/4, and SOX-2) analyzed by immunoblotting (Figure 3A). Similarly, silencing of hPaf1/PD2 resulted in a marked decrease in expression of CSC markers (CD44, and ESA) and self–renewal markers (OCT3/4, and β-Catenin) in OVCAR3 SP cells analyzed by confocal microscopy (Figure 3B). These results strongly suggest that hPaf1/PD2 is involved in the maintenance of OCSCs.

**Figure 4:**
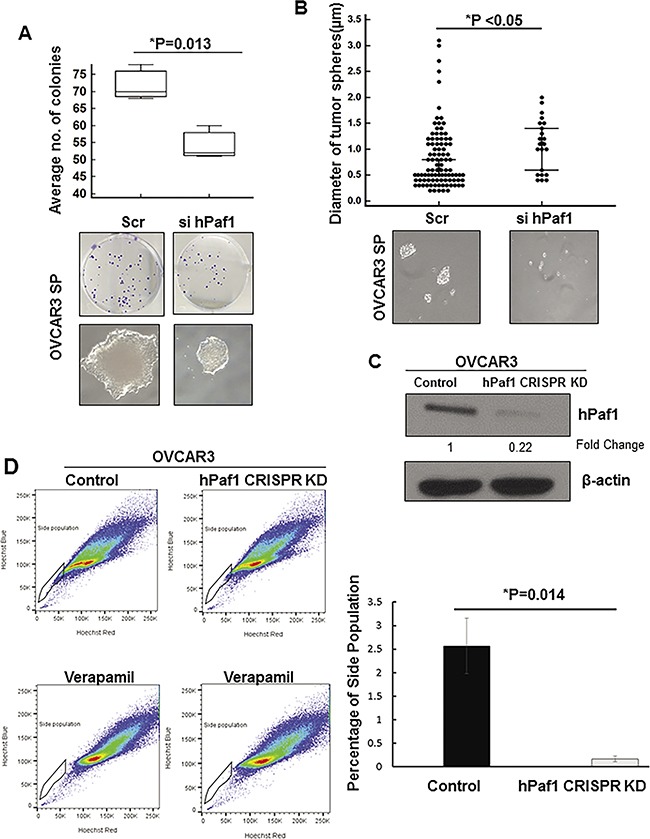
Functional studies with hPaf1/PD2 knockdown ovarian cancer stem cells **(A)** Colony formation assay was performed after transient knockdown of hPaf1/PD2. 100 pmol of hPaf1/PD2-specific siRNA or Scr siRNA was transfected in OVCAR3 SP cells. 24h after transfection, the cells were trypsinized and seeded at a density of 1000 cells/well in triplicates. The cells were allowed to form colonies and the media was changed every alternate day. After 10 days, the cells were fixed with a solution of methanol and acetone (1:1) and thereafter stained with crystal violet. Box plot indicates the average number of colonies formed with Scr siRNA or hPaf1/PD2-specific siRNA transfection. Transient knockdown of hPaf1/PD2 resulted in a significant decrease (*P* = 0.013) in number colonies formed. Upper panel consists of representative images of the wells in which cells were plated and depicting the decrease in number of colonies with hPaf1/PD2 knockdown. The lower panel depicts the decrease in size and alteration in morphology of colonies with transient knockdown of hPaf1/PD2. Knockdown of hPaf1/PD2 resulted in a loss of ‘cobblestone-like’ morphology, which is characteristic of CSCs. **(B)** Transient knockdown of hPaf1/PD2 resulted in a significant decrease (*P* < 0.05) in diameter as well as in number of tumor spheres formed. A dot plot was generated with each dot representing an individual tumor sphere of a particular diameter. The representative images of tumor sphere size in Scr siRNA and hPaf1/PD2-specific siRNA transfected cells are shown below. **(C)** Western blot analysis revealed a robust CRISPR/Cas9-mediated knockdown of hPaf1/PD2 in OVCAR3 cells. Equal amount of protein was loaded in each well. β-actin was used as a loading control. **(D)** Side population analysis showed that there was a significant decrease (*P* = 0.014) in percentage of side population cells in CRISPR/Cas9–mediated hPaf1/PD2 knockdown OVCAR3 cells as compared to untransfected control cells. The left panel depicts the representative scatter plots from the side population analysis of OVCAR3 control and OVCAR3 hPaf1/PD2 CRISPR knockdown (KD) cells with the respective verapamil controls for each sample. The graph representing the actual decrease in percentage of SP cells is presented on the right.

To analyze the functional significance of hPaf1/PD2 knockdown in OCSCs, we performed an *in vitro* tumorigenicity assay (colony formation assay), indicative of the proliferative capacity of cells, with hPaf1/PD2 silenced OVCAR3 SP cells. The cells transfected with scramble (Scr) siRNA formed significantly larger and more numerous colonies compared to hPaf1/PD2 siRNA-transfected cells (*P* = 0.013) (Figure 4A). It is important to note that silencing of hPaf1/PD2 resulted in a loss of characteristic ‘cobblestone-like’ morphology of CSCs (Figure 4A). This indicates that silencing of hPaf1/PD2 leads to loss of stemness in OCSCs, which affects their proliferative capacity.

Further, using tumor sphere assay with OVCAR3 SP cells, we observed that hPaf1/PD2 knockdown resulted in a significant decrease in the number as well as the diameter of tumor spheres (*P* < 0.05) (Figure 4B). In addition, knockdown of hPaf1/PD2 in OVCAR3 SP cells resulted in greater cell death (Supplementary Figure 3A) and downregulation of anti-apoptotic protein BCL-2 (Supplementary Figure 3B), suggesting that silencing of hPaf1/PD2 leads to greater apoptosis of SP cells. These results indicate that hPaf1/PD2 plays a role in the maintenance of OCSCs and that knockdown of hPaf1/PD2 severely affects the CSC phenotype.

### CRISPR/Cas9–mediated knockdown of hPaf1/PD2 decreases the ovarian cancer stem cell population

Since our experiments were uncovering a role of hPaf1/PD2 in maintenance of the OCSC phenotype, we next investigated the impact of loss of hPaf1/PD2 on OCSC population (SP). Utilizing the CRISPR/Cas9 system, which has emerged as an efficient tool to modulate gene expression [25, 26], we knocked down hPaf1/PD2 in OVCAR3 using guide RNA specific for hPaf1/PD2. We were unable to generate single cell clones with complete knockout of hPaf1/PD2 as these single cell clones did not survive, indicating that knockout of hPaf1/PD2 might be lethal. However, immunoblotting revealed a robust knockdown of hPaf1/PD2 in pooled population of transfected OVCAR3 cells as compared to control cells that were not transfected (Figure 4C). SP analysis in the pooled population revealed that there was a significant decrease in proportion of SP cells in OVCAR3 cells with CRISPR/Cas9-mediated hPaf1/PD2 knockdown as compared to control cells (*P* = 0.014) (Figure 4D), further corroborating the role of hPaf1/PD2 in OCSC maintenance.

### hPaf1/PD2 interacts with OCT3/4 for the maintenance of ovarian cancer stemness

**Figure 5:**
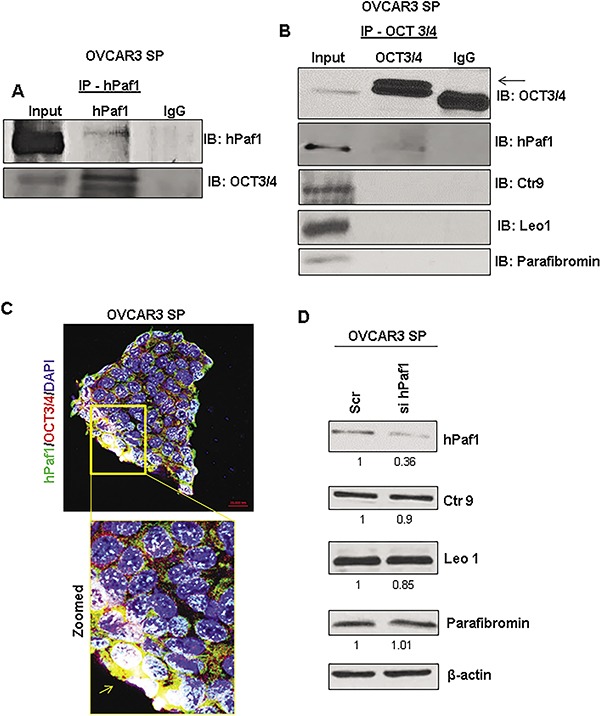
PAF Complex independent interaction of hPaf1/PD2 with OCT 3/4 in ovarian cancer stem cells (**A**) Co-immunoprecipitation assay showed that hPaf1/PD2 interacts with OCT3/4. hPaf1/PD2 antibody was used for pulldown and immunoprecipitates were probed with OCT3/4 antibody. Absence of non-specific binding was confirmed by including an IgG control. (**B**) We also performed reciprocal co-immunoprecipitation assay to pull down OCT3/4 and probed with hPaf1/PD2 antibody. We did not observe any interaction of OCT3/4 with other PAF complex components such as Leo1, Ctr9 and Parafibromin. Absence of non-specific binding was confirmed by including an IgG control. (**C**) Co-localization of hPaf1/PD2 with OCT3/4 was also observed using confocal microscopy. The box indicates a zoomed image depicting perinuclear and nuclear co-localization of hPaf1/PD2 with OCT3/4. (**D**) Western blotting analysis revealed that there was no change in expression of other PAF complex components such as Ctr9, Leo1, and Parafibromin on knockdown of hPaf1/PD2. Equal amount of protein was loaded in each well. β-actin was used as a loading control.

It has been reported that hPaf1/PD2 is involved in the maintenance of mouse embryonic stem cells through its interaction with OCT3/4 [20]. Another study has demonstrated the transcriptional regulation of OCT3/4 by members of the PAF complex in mouse embryonic stem cells [27]. Moreover, OCT3/4 is a gatekeeper for the process of self-renewal in stem cells [28, 29]. Since OCT3/4 showed co-localization and altered expression along with hPaf1/PD2 in SP cells, we sought to analyze the interaction between these two proteins. Reciprocal co-immunoprecipitation assay showed a clear interaction between hPaf1/PD2 and OCT3/4 in OVACR3 SP cells (Figure 5A and Figure 5B). In addition, confocal microscopy revealed co-localization of OCT3/4 with hPaf1/PD2 in the nuclear and the peri-nuclear area (Figure 5C). These results strongly suggest the involvement of hPaf1/PD2 in maintenance of the self–renewal characteristics of OCSCs through its interaction with OCT3/4.

### hPaf1/PD2 functions independently to maintain self-renewal of OCSCs

Previous studies from our lab have reported that hPaf1/PD2 can function independent of the PAF complex in stem cells despite being part of the PAF complex (PAFC) [19, 20]. To determine whether hPaf1/PD2 has PAFC independent functions in the context of OCSCs, we analyzed the expression of other PAFC components in hPaf1/PD2-silenced OVCAR3 SP cells through immunoblotting. There was no significant change in expression of other complex components (Leo1, Ctr9, and Parafibromin) on knockdown of hPaf1/PD2 (Figure 5D), suggesting uncoordinated expression of PAFC components in OCSCs. We further found that only hPaf1/PD2 interacts with OCT3/4, and there is no interaction between OCT3/4 and other PAFC components (Figure 5B). These results suggest that hPaf1/PD2 functions independently of the PAFC in the maintenance of self-renewal of OCSCs.

### hPaf1/PD2 is differentially co-expressed with self-renewal marker OCT3/4 and CSC marker ESA in different stages of ovarian cancer, compared to normal ovarian tissue

**Figure 6:**
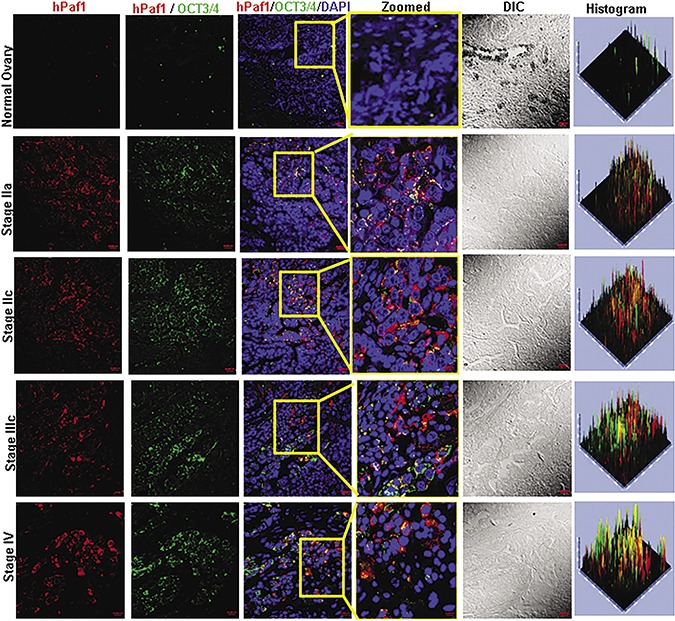
Expression of hPaf1/PD2 and self-renewal marker OCT3/4 in human ovarian cancer tissue array Confocal microscopic analysis showed a significantly higher expression of hPaf1/PD2 as well as OCT3/4 in human ovarian cancer tissues compared to normal ovarian tissues. The expression of hPaf1/PD2 with OCT3/4 was highly coincident in ovarian cancer tissues; however; there was no co-localization in normal ovarian tissues. The highlighted box shows the zoomed image.

It is well known that OCT3/4 is the master regulator of pluripotency and is important for the maintenance of the self-renewal process of SCs [28, 29]. Here, we analyzed the expression of hPaf1/PD2 with OCT3/4 in different stages of OC, as well as normal ovarian tissues, using confocal microscopic analysis of spotted tissue array (Figure 6). There was a minimal expression of hPaf1/PD2 in normal ovarian tissues; however, in the various stages of OC, there was a significantly higher expression of hPaf1/PD2 as well as OCT3/4, and the expression was highly coincident. We also performed dual confocal microscopic staining for hPaf1/PD2 and CSC marker ESA using serial sections of previously used spotted tissue array. We found that there was a significant co-localization and overexpression of hPaf1/PD2 with ESA in various OC stages compared to normal tissues (Supplementary Figure 4). The preferential co-localization of hPaf1/PD2 with OCT3/4 and ESA in different stages of OC compared to normal ovarian tissues confirms the presence of OCSCs in OC tissues.

## DISCUSSION

Emerging evidence demonstrates that cancer stem cells (CSCs) are a small population of specialized cells thought to contribute to metastasis, drug resistance, and tumor relapse [30]. Moreover, recent findings have focused on targeting these CSCs using molecularly targeted agents. To date, several CSC markers, such as CD44, CD133, CD24, ALDH1, and c-kit, have been identified for OCSCs [3, 31]. However, CSC maintenance drivers are poorly understood, making it difficult to target this population. The majority of OC patients have advanced or metastatic disease at the time of diagnosis, and the standard of care consists of platinum based chemotherapy with cytoreductive surgery in appropriate surgical candidates. Despite significant improvements in the overall survival of OC patients with conventional therapy, more than 85% of cases relapse. This is in part due to the development of therapy resistance [32], which can be mediated by CSCs. Therefore, delineating the molecular mechanisms underlying tumor relapse and therapy resistance would pave avenues to devise effective therapeutic strategies. In this study, we have investigated the role of hPaf1/PD2 inthe maintenance of self-renewal of OCSCs.

First, we confirmed the expression of hPaf1/PD2 in clinical samples. Our immunohistochemical analysis revealed that only a small number of patient tissues exhibited moderate-to-strongly positive hPaf1/PD2 expression (32.4%), whereas the majority of patients’ tissues showed basal to negative expression (67.6%) in epithelial OC cells. Previous studies have shown that hPaf1/PD2 is involved in tumorigenesis and metastasis [9, 18] and in regulating post-translational modifications such as histone methylation and chromatin remodeling in pancreatic cancer cells [12]. Our recent studies have also shown an association between hPaf1/PD2 with CSCs and disease aggressiveness [18, 19]. This is the first report to show the associative and expressional variation of hPaf1/PD2 in OC patient tissues. Our results demonstrate that hPaf1/PD2 is overexpressed only in a subpopulation of cells within the tumor, and that its degree of expression varies from basal to moderate to strong in this subpopulation of cells. Thus, hPaf1/PD2 may be important for a specific population of cells such as CSCs, that can be further exploited for therapeutic and functional studies.

Further, in addition to human primary pancreatic and metastatic tumors, the 19q13 chromosomal locus that contains hPaf1/PD2 gene has been shown to be amplified in OC cells [33]. We thus wanted to examine the relevance of hPaf1/PD2 in *in vitro* and *in vivo* settings of OC. Therefore, the second objective of our study was to isolate side population (SP) and non-SP (NSP) cells from the A2780 and OVCAR3 cells and validate the expression of CSC markers. SP cells isolated from both cell lines showed increased expression of CSC markers (CD133, CD44, CD24, ESA, and Lgr5), and self-renewal proteins (β-Catenin, OCT3/4, SHH, SOX-2, SOX-9, and HER2), along with hPaf1/PD2, as compared to NSP cells. Among all these global CSC markers, we found hPaf1/PD2 to be significantly overexpressed in specific cells of OC tissues and CSCs. The expressional co-relation of hPaf1/PD2 with CSC and self-renewal markers in SP cells suggested that the hPaf1/PD2 overexpressing SP cells are putative OCSCs. Similar findings have been reported by Bailey *et al*. for pancreatic CSCs, where they documented that DCLK1 high cells/acetylated tubulin high cells were also positive for CSC markers such as CD133 or CD24/CD44/ESA [34]. Previously, various CSC markers and self-renewal proteins such as CD133, CD44, ALDH1, ESA, β-Catenin, and SHH were found to be overexpressed in various cancers, including, but not limited to, pancreatic, head and neck cancer, prostate, breast, ovary, and lung cancer [3, 6, 35–39]. We examined the hPaf1/PD2 overexpressing SP cells for *in vitro* tumor sphere-forming ability and found that hPaf1/PD2 expressing SP cells displayed greater number of tumor spheres, compared to NSP cells isolated from the same parental OC cells. Several studies have documented the ability of CD133^+^ and CD44^+^ CSCs to grow faster and to elicit a highly aggressive nature compared to their corresponding negative population or NSP cells [40, 41].

**Figure 7:**
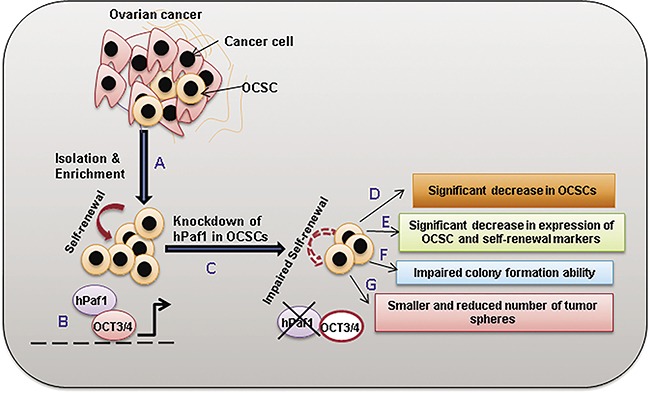
Overall scheme depicting the role of hPaf1/PD2 in the maintenance of OCSCs Ovarian cancer is heterogeneous, consisting of multiple different cell types including but not limited to cancer cells and cancer stem cells. **(A)** OCSCs were isolated via Hoechst33342 staining through flow cytometry and were enriched via culture in CSC-specific media in combination with cisplatin treatment. **(B)** OCSCs possess the property of self-renewal which is maintained by hPaf1/PD2 through its interaction with OCT3/4. This interaction possibly regulates the transcription of downstream self-renewal genes in OCSCs (indicated with the dotted line). **(C)** Knockdown of hPaf1/PD2 was performed in OCSCs and its functional implications were analyzed. Loss of hPaf1/PD2 caused **(D)** a significant decrease in proportion of OCSCs; **(E)** downregulation of OCT3/4 and other CSC and self-renewal markers; **(F)** impairment of colony formation; and **(G)** reduction in tumor sphere formation ability of OCSCs; in turn resulting in impaired self-renewal capacity (indicated with the dotted arrow). Overall, hPaf1/PD2 could be an important target to modulate the self-renewal capacity of OCSCs via its interaction with OCT3/4.

Third, we investigated the biological and phenotypic effects of hPaf1/PD2 knockdown on the OCSCs. We observed a marked decrease in expression of CSC markers, including CD44, CD133, and ESA, as well as self-renewal proteins such as SHH, β-Catenin, OCT3/4, and SOX-2 following transient knockdown of hPaf1/PD2 in OVCAR3 SP cells. Previously, Vaz *et al*. had demonstrated that CD133 and MDR2 expression is reduced upon knockdown of hPaf1/PD2 in pancreatic CSCs [19]. Of note, through this report we found that expression of self-renewal and established CSC markers could be modulated upon hPaf1/PD2 silencing. We also found that knockdown of hPaf1/PD2 in SP cells reduced the number of colonies and tumor spheres formed compared to their respective controls. These data corroborate the critical role of hPaf1/PD2 in self-renewal and the tumorigenic property of OCSCs. A similar observation has been made in another study, wherein siRNA directed against casein kinase 2 resulted in the decreased tumor sphere-forming efficiency of SKOV3-derived sphere-forming cells, indicating impaired self-renewal abilities [42]. Further, CRISPR/Cas9-mediated knockdown of hPaf1/PD2 resulted in a significant decrease in SP in OC cells; further corroborating the role of hPaf1/PD2 in OCSC maintenance. Our knockdown studies also alluded to the underlying molecular mechanisms of the role of hPaf1/PD2 in OCSCs (Figure 7). Specifically, knockdown of hPaf1/PD2 resulted in a decrease of protein expression of OCT3/4, which has previously been shown to be associated with maintenance of self-renewal of mouse embryonic stem cells [20, 43] and CSC [44, 45]. Indeed, our studies showed that hPaf1/PD2 can physically interact with OCT3/4 and participate in maintenance of OC stemness. On the other hand, we were not able to detect any possible interaction of OCT3/4 with other PAF complex components such as Ctr9, Leo1, and Parafibromin. This suggested a complex-independent function for hPaf1/PD2 on OCT3/4 in OCSCs.

Several studies have shown the importance of OCT3/4 in maintaining the self-renewal property of stem cells and that it is the master regulator of pluripotency [28, 29]. Moreover, in addition to being expressed in a large number of cancer cells, OCT3/4 has been reported to be essential for eliciting CSC-related properties such as self-renewal and CSC maintenance. Recent studies have demonstrated OCT3/4 to be highly expressed in liver, lung, and bladder CSCs and that OCT3/4 participates in self-renewal, maintenance of CSCs, and regulation of tumor progression and metastasis [44, 46, 47]. The interaction of OCT3/4 with hPaf1/PD2 reported here alludes to a mechanistic role of hPaf1/PD2 in regulating the self-renewal pathway of OCSCs via its interaction with OCT3/4.

Finally, we demonstrated that hPaf1/PD2 is significantly co-overexpressed with ESA and OCT3/4 in OC tissues compared to normal ovary tissues. In this report, we have shown that hPaf1/PD2 co-localizes with ESA, CD44, SHH, and OCT3/4 and that the same markers were decreased upon hPaf1/PD2 knockdown. This implies a functional and phenotypical relevance of these established CSC markers with hPaf1/PD2 in OC. A study by Klun *et al*. has shown putative CSC markers, such as Nanog, SOX-2, and SSEA-4 to be expressed in the borderline and high-grade OC specimens [48], suggesting a critical role for these CSC markers in OC.

In summary, we observed that hPaf1/PD2 is differentially expressed in human OC tissues and is overexpressed in OCSCs. Knockdown of hPaf1/PD2 affects the OCSC phenotype as it results in decreased expression of CSC and self-renewal markers, reduced colonies and tumor spheres *in vitro*, and decrease in the proportion of OCSCs. Moreover, hPaf1/PD2 specifically interacts with OCT3/4 to elicit CSC function and in clinical settings, hPaf1/PD2 co-expresses with OCT3/4 and ESA in all stages of human OC with no such co-expression in normal ovary specimens. It is quite possible that OCSCs use each of these stem cell markers with hPaf1/PD2 in a context-dependent manner. In future studies, we plan to examine the mechanistic relevance of hPaf1/PD2 with other CSC maintenance markers. Our data indicate that hPaf1/PD2 is a novel molecule that might be a maintenance driver for OCSCs, and that it is responsible for OCSC maintenance through its interaction with OCT3/4 (Figure 7). Our study also strongly suggests that hPaf1/PD2 is a novel protein that can be employed for therapeutic targeting of OCSCs.

## MATERIALS AND METHODS

### Cell culture

Human OC cell lines OVCAR3 and A2780 were obtained from ATCC. A2780 was cultured in DMEM media (HyClone Laboratories, Logan, UT, USA) supplemented with 10% fetal bovine serum (FBS) (Sigma-Aldrich, St Louis, MO, USA) and 1% penicillin-streptomycin solution (Sigma). OVCAR3 was cultured in RPMI media (HyClone) supplemented with 20% FBS, 2 mM glutamine (Sigma), 1.5 g/L sodium bicarbonate (HyClone), 2.5 g/L dextrose (Sigma), 10 mM HEPES (Sigma), 10 mM sodium pyruvate (Sigma), 0.01 mg/ml bovine insulin (Sigma), 100 U/ml penicillin and 10 μg/ml streptomycin (Sigma). Cells were subcultured by trypsin-EDTA treatment with complete medium changed every other day.

### Human ovarian cancer tissues and tissue array

Paraffin embedded human OC tissues (*n* = 37) were obtained from the UNMC tissue bank. Samples were obtained following protocol approval by the Institutional Review Board (IRB) at the University of Nebraska Medical Center in Omaha, Nebraska. OC tissue array (OV243), which consists of normal ovary tissues (*n* = 6) and tissues of different OC stages (*n* = 18) was obtained from US Biomax (Rockville, MD, USA).

### Isolation of side population (SP) and non-side population (NSP) from cancer cell lines

Side population (SP) cells or putative CSCs were isolated using flow sorting following Hoechst 33342 (AnaSpec Inc., Fremont, CA, USA) staining as described previously [19]. Gating to identify the characteristic SP was aided with the inclusion of a Verapamil (Sigma) control, a calcium channel blocker that reverses the drug resistance phenotype. The remaining population with higher intensity of Hoechst stain that fell outside the gate was designated as the ‘non-side population’ (NSP).

### CSC-specific cell culture

Stem cell-specific medium was used for the culture of isolated SP and NSP cells as described previously [19]. SP and NSP fractions obtained via flow sorting from OVCAR3 and A2780 were grown in RPMI, supplemented with 20% FBS and other OVCAR3 supplements and DMEM with 10% FBS, respectively, for a day to allow the cells to acclimatise. The cells were then transferred to a stem cell-specific medium. SP cells were treated with 2 μM cisplatin (IC20) for enriching the CSC population.

### Immunoblot assay

OVCAR3 and A2780 cell lines were processed for protein isolation and Western blotting using standard procedures, as described previously [19]. The following primary antibodies were used: anti-hPaf1/PD2, anti-Leo1, anti-Parafibromin, anti-Ctr9 (Bethyl Laboratories, Montgomery, TX, USA); anti-OCT3/4, anti-SOX-2, anti-CD24, anti-ESA, anti-SHH, anti-HER2 (Santa Cruz Biotechnology, Dallas, TX, USA); anti-CD44 (Cell signalling Technology, Danvers, MA, USA); anti-CD133 (Abnova, Walnut, CA, USA); anti–β-Catenin (Sigma); and anti-Lgr5, anti-SOX-9 (Abcam, Cambridge, MA, USA) overnight at 4°C. β-actin was used as a loading control. The band intensity was quantified using ImageJ and the normalization was performed as described previously [18].

### Immunohistochemistry

Immunohistochemistry (IHC) analysis was performed as described previously [49]. We used our in-house generated, anti-PD2 mouse monoclonal antibody at a dilution of 1:500 and performed overnight incubation [9]. hPaf1/PD2 expression in human ovarian tissues was scored by a UNMC pathologist using double blind conditions. Each sample was given a composite score (CS) based on intensity and extent of tissue staining. Intensity was graded on a four-point scale of 0–3 (0− no staining, 1+ weakly positive, 2+ moderately positive and 3+ strongly positive). The extent of hPaf1/PD2-positive staining in human OC tissues was scored as actual percentages. A composite score (CS) was calculated by multiplying the intensity and positivity, which ranged between 0 and 3.

### Immunofluorescence analysis

Cells were plated, fixed and processed as described previously [19]. Primary antibodies specific for rabbit-hPaf1/PD2 (1:100 in PBS), rabbit-SHH (1:100), mouse-OCT3/4 (1:100), rabbit-β-Catenin (1:100), mouse-CD44 (1:250) and rabbit-ESA (1:1500) were used with a 4 h incubation for cells. For the tissue array, we followed the same procedure mentioned previously [49], but incubation with primary antibodies was performed overnight at 4°C. Following primary antibody incubation, the cells and tissue sections were processed using standard procedures as described previously [19].

### Knockdown of hPaf1/PD2 using specific siRNA

Transient knockdown of hPaf1/PD2 was performed using hPaf1/PD2 siRNA (Santa Cruz Biotechnology), which is a pool of 3 target-specific 19–25 nt siRNAs. OVCAR3 SP cells were plated in a 6 well plate at a concentration of 0.6 million/well. On the following day, the cells were serum starved for 4 h, and then transfected with hPaf1/PD2 siRNA or non-targeting control siRNA (scramble siRNA) at a concentration of 100 pmol/ well. Serum containing medium was added to the cells 4 hours after transfection. The medium was changed every 24 h and lysates were collected 72 h after transfection.

### Colony formation assay (clonogenic assay) with hPaf1/PD2 knockdown in SP cells

OVCAR3 SP cells were transfected with 100 pmol/ well of Paf1 siRNA or scramble (Scr) siRNA as described in the previous section. 24 h later, the cells were trypsinized and seeded at a density of 1000 cells/well in a 6 well plate in triplicates. The cells were cultured in CSC-specific media, with media changed once in two days. After 2 weeks of growth, cells were imaged using the Motic AE 2000 microscope with an attached Moticam5 camera to capture the gross morphological variations between hPaf1/PD2 knockdown cells and scramble siRNA transfected cells. Thereafter, the cells were fixed with 100% methanol and stained with crystal violet stain (0.1%, w/v in 20 nm 4-morpholinepropanesulfonic acid; Sigma) before the colonies started to merge. The staining was quantified using ImageJ and the graph was plotted using MedCalc software.

### Tumor sphere assay

OVCAR3 SP cells transfected with hPaf1/PD2 siRNA or Scr siRNA were seeded in triplicates in a 24 well non-adherent plate (Corning Inc., Corning, New York, USA) in CSC-specific media at a concentration of 100 cells/well. The cells in suspension culture were observed under the microscope and fresh media was added every alternate day without removing the existing media. A week later, multiple images were taken per well for different fields of view. The diameter of each tumor sphere was measured using Motic Images Plus 2.0 ML software; the dot plot depicting the diameter of tumor sphere in hPaf1/PD2 knockdown cells and Scr siRNA treated cells was plotted using MedCalc software.

### CRISPR/Cas9-mediated hPaf1/PD2 knockdown

hPaf1/PD2 knockdown in OVCAR3 cells was performed using CRISPR/Cas9 system [50]. Briefly, cells were transfected with hPaf1/PD2 guide RNA (5’- ACCTACCGCATCGACCCCAA -3’) containing CRISPR/Cas9 vector (pSpCas9 BB-2A-GFP PX458) (Genescript, Piscataway, NJ, USA). 72 h later, GFP positive cells were isolated and the pooled population was collected in a 12 well plate by flow sorting. Cells were allowed to grow in to colonies, which were then analyzed for expression of hPaf1/PD2 by immunoblot analysis.

### Immunoprecipitation analysis

OVCAR3 SP cells were treated with an amine reactive protein cross linker, DSP ((dithiobis (succinimidyl propionate)) (ThermoFisher Scientific, Waltham, MA, USA), to stabilize the interaction according to manufacturer's instructions. The lysate was collected in a non-denaturing immunoprecipitation buffer (20 mM Tris, pH 7.5, 200 mM NaCl, 1% NP-40, 10% Glycerol, 1 mM DTT). Immunoprecipitation was performed with anti-hPaf1/PD2 (rabbit polyclonal, Bethyl Laboratories) and anti-OCT3/4 (mouse monoclonal, Santa Cruz Biotechnology) antibodies as described previously [12]. The immunoprecipitates or total cell lysates were transferred onto the PVDF membrane after being resolved on 10% SDS PAGE, and thereafter were incubated overnight at 4°C with primary antibodies (anti-hPaf1/PD2, anti-OCT3/4, anti-Leo1, anti-Ctr9, anti-parafibromin).

### Statistical analysis

Student *t-test* was used to determine the statistical significance between control and hPaf1/PD2 knockdown group in all the experiments pertaining to this study. Statistical analysis and generation of graphs were performed using MedCalc software. *P value* of less than 0.05 was considered to be statistically significant. Error bars were given on the basis of calculated standard error values.

## SUPPLEMENTARY MATERIALS FIGURES AND TABLE


