# Supplementary material for: hPaf1/PD2 interacts with OCT3/4 to promote self-renewal of ovarian cancer stem cells
Source: Oncotarget. 2017 Jan 20;8(9):14806–20. doi: 10.18632/oncotarget.14775 (PMC5362445; doi:10.18632/oncotarget.14775)
Supplement: Supplementary file 1 [file oncotarget-08-14806-s001.pdf]

hPaf1/PD2 interacts with OCT3/4 to promote self-renewal of ovarian cancer stem cells

Supplementary Materials

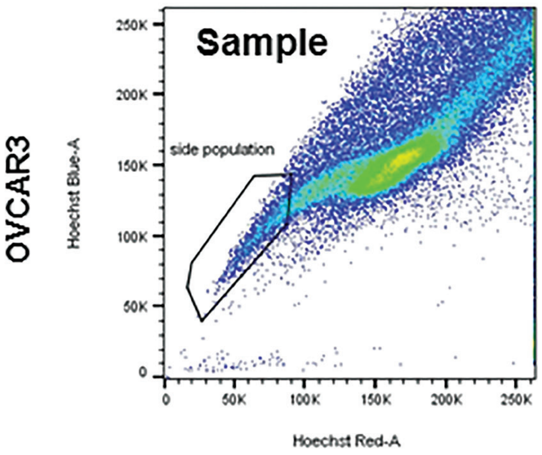

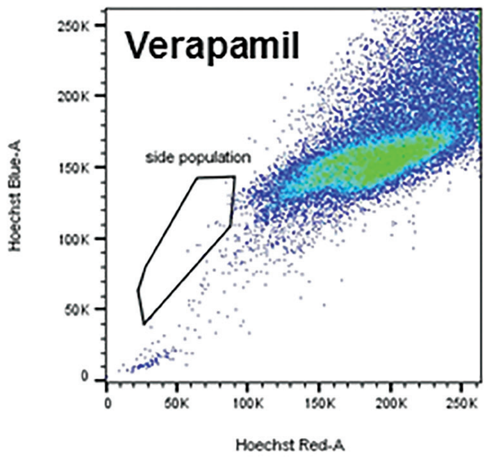

Tube: sp_PI

Population	#Events	%Parent	%Total
All Events	108,786	####	100.0
gate	99,206	91.2	91.2
sp	3,070	3.1	2.8
non sp	2,144	2.2	2.0
dead	1,134	1.1	1.0

Tube: verapamil

Population	#Events	%Parent	%Total
All Events	73,799	####	100.0
gate	62,130	84.2	84.2
sp	34	0.1	0.0
non sp	909	1.5	1.2
dead	1	0.0	0.0

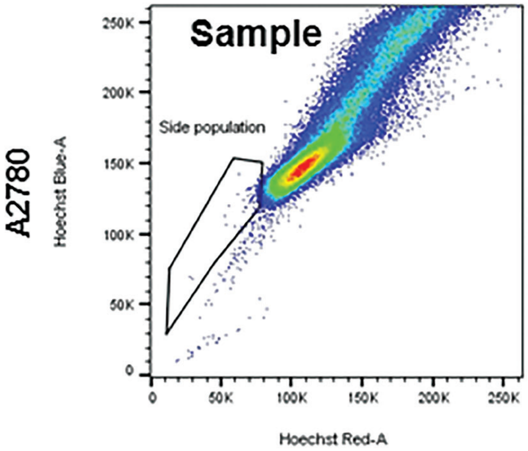

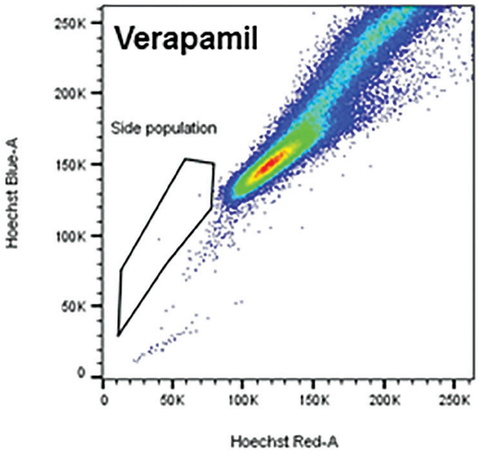

Tube: sample

Population	#Events	%Parent	%Total
All Events	132,855	####	100.0
GATE	106,104	79.9	79.9
DD GATE	100,984	95.2	76.0
DD GATE 2	99,783	98.8	75.1
SP	559	0.6	0.4

Tube: verapamil_002

Population	#Events	%Parent	%Total
All Events	127,458	####	100.0
GATE	103,683	81.3	81.3
DD GATE	100,991	97.4	79.2
DD GATE 2	99,806	98.8	78.3
SP	16	0.0	0.0

Supplementary Figure 1: Isolation of CSCs from ovarian cancer cells lines OVCAR3 and A2780. Flow cytometric sorting of side population from OVCAR3 and A2780 using Hoechst 33342 dye efflux.

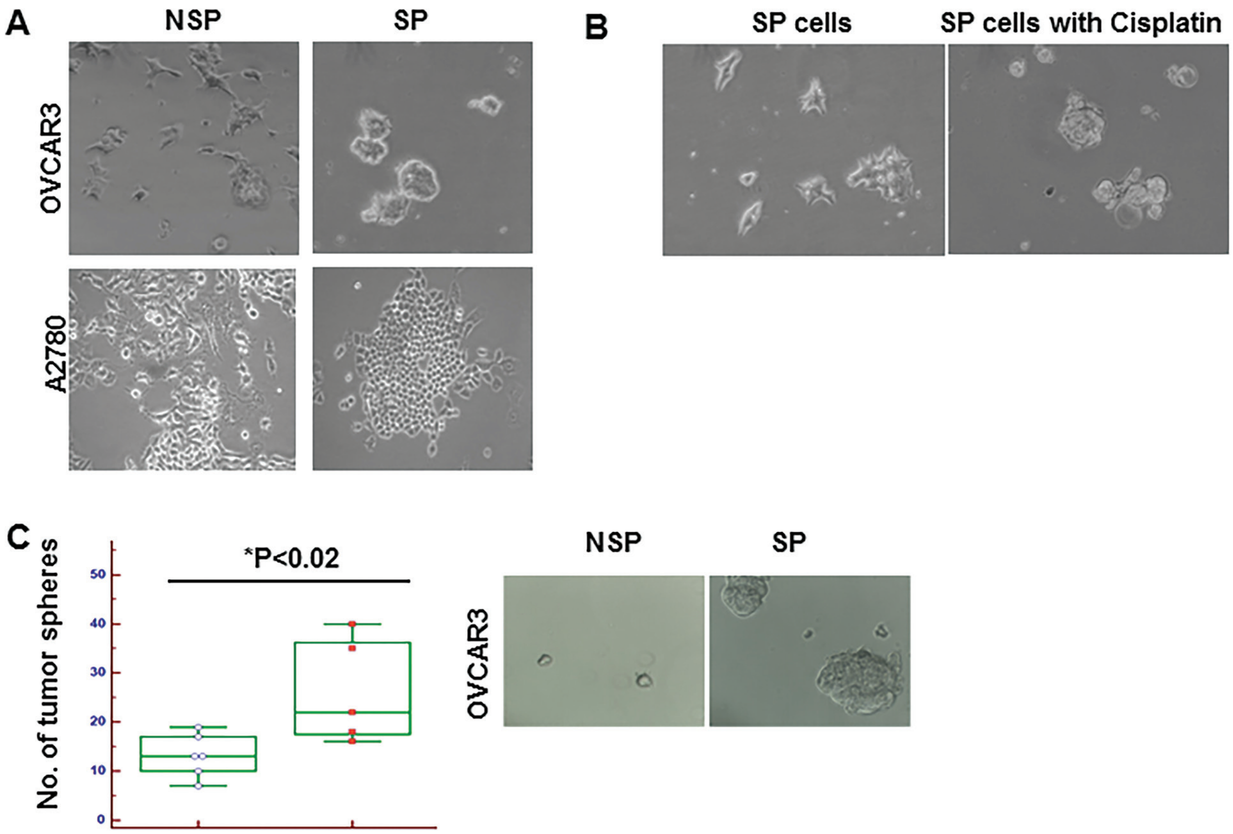

Supplementary Figure 2: Characterization of isolated ovarian cancer stem cells. (**A**) SP cells isolated from OVCAR3 and A2780 and cultured in CSC-specific media showed 'cobblestone-like morphology', whereas NSP cells appeared similar to differentiated cells. (**B**) SP cells treated with cisplatin (IC20 = 2 μM) exhibited a more 'cobblestone-like' morphology compared to SP cells without cisplatin treatment, indicating enrichment of CSCs by cisplatin. (**C**) Box plot depicting the number of tumor spheres formed by NSP cells and SP cells. The images on the right represent the size and number of tumor spheres formed by NSP and SP cells isolated from OVCAR3.

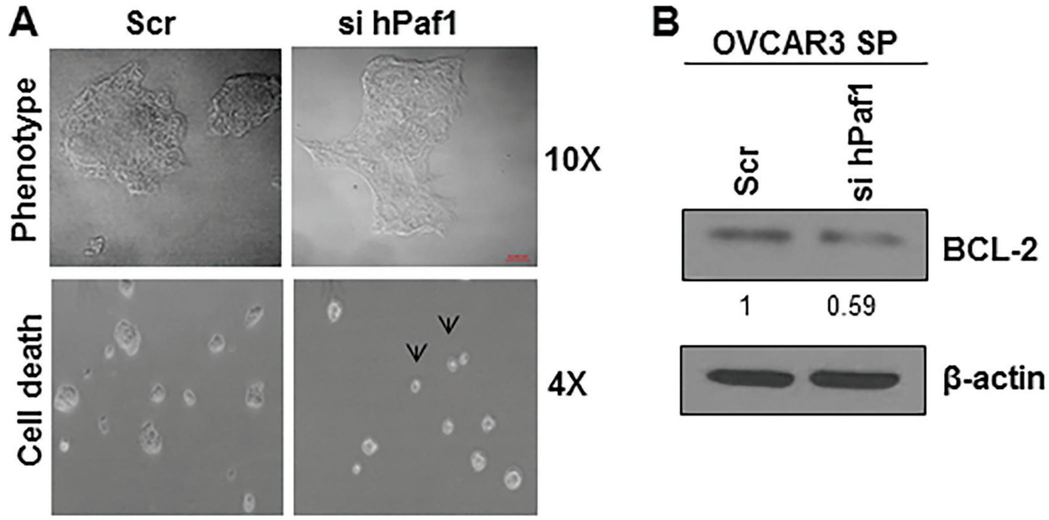

Supplementary Figure 3: Effect of knockdown of hPaf1/PD2 in OCSCs. (**A**) Cells in which hPaf1/PD2 was knocked down showed more cell death than cells transfected with Scr siRNA. (**B**) hPaf1/PD2 silenced cells exhibited lower expression of anti-apoptotic protein BCL-2 by Western blotting, indicating that they were more susceptible to cell death. Equal amount of protein was loaded in each well. β-actin was used as a loading control.

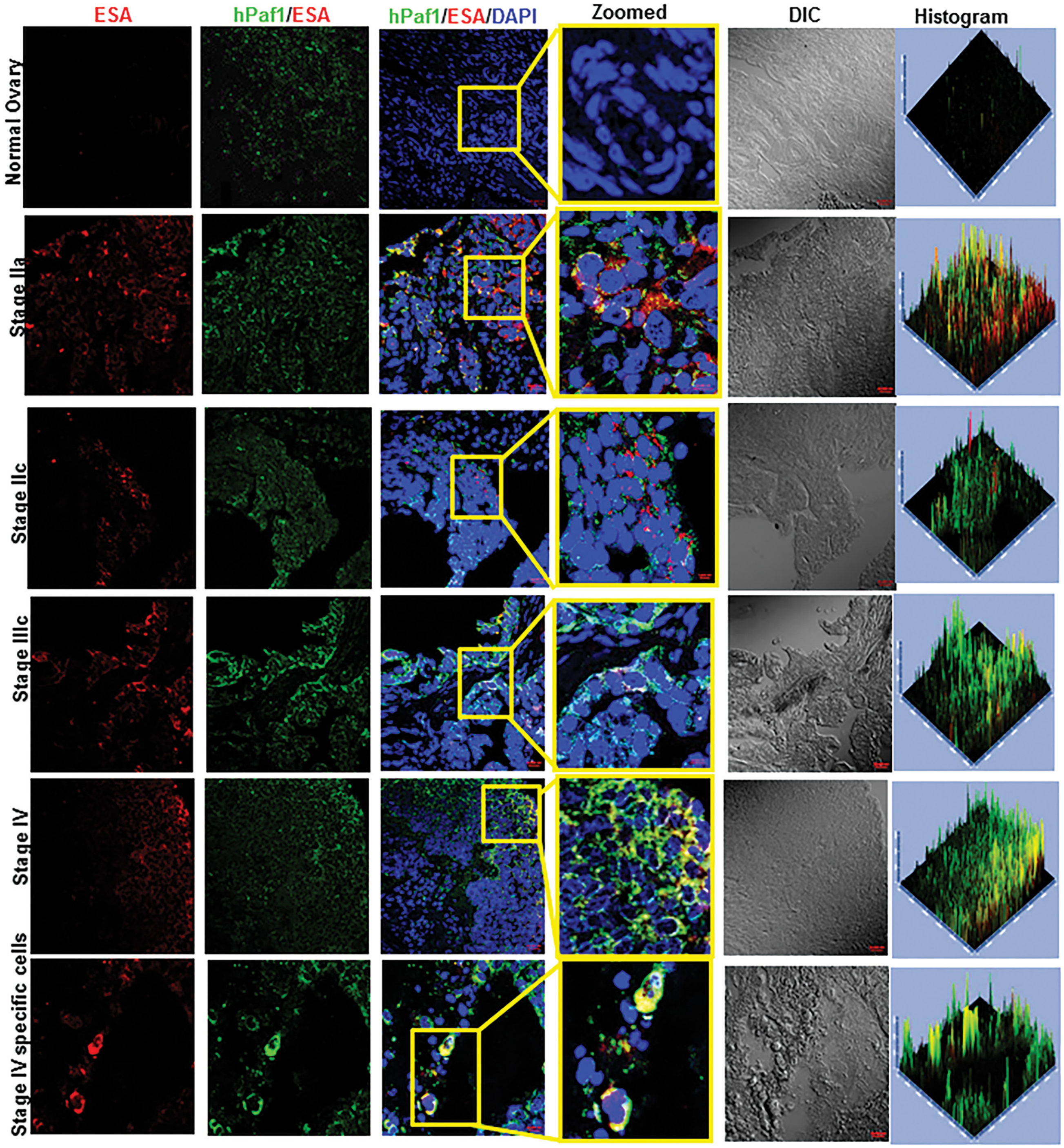

Supplementary Figure 4: Expression of hPaf1/PD2 and CSC marker ESA in ovarian cancer tissue array. Expression of hPaf1/PD2 and CSC marker ESA was analyzed on US Biomax ovarian cancer tissue array using immunofluorescence. There was significant over-expression and co-localization of hPaf1/PD2 along with ESA in different stages of ovarian cancer. There was minimal expression and no co-localization of hPaf1/PD2 with ESA in normal ovarian tissues. The highlighted box shows the zoomed image.

Supplementary Table 1: Composite score for hPaf1/PD2 expression in human OC tissues categorized as areas with low and high intensity of staining

Composite score for areas with low intensity	Composite score for areas with high intensity
0.05	0.6
0.325	0.65
0.1	1.4
0.25	0.5
0.4	0.6
0.05	1.5
0.2	1.2
0.2	0.8
0.1	0.2
0.02	0.04
0.3	0.3
0.55	0.5
